# An emergentist perspective on the origin of number sense

**DOI:** 10.1098/rstb.2017.0043

**Published:** 2018-01-01

**Authors:** Marco Zorzi, Alberto Testolin

**Affiliations:** 1 Department of General Psychology and Padova Neuroscience Center, University of Padova, Via Venezia 12, Padova 35131, Italy; 2 IRCCS San Camillo Hospital Foundation, Venice-Lido, Italy

**Keywords:** number sense, numerosity perception, numerical development, computational modelling, deep learning, artificial neural networks

## Abstract

The finding that human infants and many other animal species are sensitive to numerical quantity has been widely interpreted as evidence for evolved, biologically determined numerical capacities across unrelated species, thereby supporting a ‘nativist’ stance on the origin of number sense. Here, we tackle this issue within the ‘emergentist’ perspective provided by artificial neural network models, and we build on computer simulations to discuss two different approaches to think about the innateness of number sense. The first, illustrated by artificial life simulations, shows that numerical abilities can be supported by domain-specific representations emerging from evolutionary pressure. The second assumes that numerical representations need not be genetically pre-determined but can emerge from the interplay between innate architectural constraints and domain-general learning mechanisms, instantiated in deep learning simulations. We show that deep neural networks endowed with basic visuospatial processing exhibit a remarkable performance in numerosity discrimination before any experience-dependent learning, whereas unsupervised sensory experience with visual sets leads to subsequent improvement of number acuity and reduces the influence of continuous visual cues. The emergent neuronal code for numbers in the model includes both numerosity-sensitive (summation coding) and numerosity-selective response profiles, closely mirroring those found in monkey intraparietal neurons. We conclude that a form of innatism based on architectural and learning biases is a fruitful approach to understanding the origin and development of number sense.

This article is part of a discussion meeting issue ‘The origins of numerical abilities'.

## 1. Introduction

It is widely believed that mathematical learning is rooted into a phylogenetically ancient ‘number sense’ that humans share with many animal species [1,2]. Perceiving the number of objects is a key aspect of the number sense and it is highly adaptive for survival [3,4]. Visual numerosity appears to be extracted directly and spontaneously from vision [5,6] by a specialized mechanism that yields an approximate representation of numerical quantity, the approximate number system (ANS) [7]. The ANS representation can be conceived as a distribution of activation on a putative ‘mental number line’, where the overlap between distributions of activation increases with numerical magnitude due to either scalar variability or compression of the scale [8,9]. Accordingly, discrimination between two numerosities is modulated by their numerical ratio, thereby obeying Weber's Law, and this ratio-dependent effect in numerosity comparison is considered to be a primary signature of the ANS. The striking similarity in performance between human and non-human primates has suggested phylogenetic continuity of the ANS [10], as also indicated by the shared neural correlates found in the intraparietal sulcus of the primate brain [11,12].

The ability to discriminate between numerosities, known as number acuity, improves throughout childhood [13,14]. Human babies seem to be able, since their first hours of life, to discriminate the numerosity of object sets if the ratio is at least 1 : 3 [15]. Dramatic changes in number acuity have been observed within the first years of life: for instance, six-month-old infants can reliably discriminate between sets with a ratio of 1 : 2 but fail with a 2 : 3 ratio, which is instead discriminated by 10-month-olds [16,17]. The 2.5-year-old toddlers discriminate a ratio of 3 : 4 [18]. Changes in number acuity are thought to index the representational precision of the ANS, and it is widely believed that the latter is foundational to the subsequent acquisition of formal numerical competences. Indeed, individual number acuity has been linked to mathematical achievement [19–21] (for a meta-analysis, see [22]) and it has been showed to be impaired in dyscalculia [14,23].

In the present article, we discuss the origin of number sense within the emergentist framework provided by artificial neural network models [24]. The emergentist approach to cognition [25] assumes that the structure seen in overt behaviour and its patterns of change (e.g. during development) reflect the operation of subcognitive processes, such as propagation of activation and inhibition among neurons and adjustment of strengths of connections between them. In the context of the visual number sense (i.e. numerosity perception), the crucial question is what kind of biological constraints lead to its emergence (for a broader discussion, also see [26]). Is the number sense innate? We show below that connectionist simulations with artificial neural networks can provide a fresh perspective on this debate. Elman *et al*. [27] have thoroughly discussed the issue of innateness in the context of connectionism. Here, we focus on their observation that there are two different ways to think about innateness, which also readily apply to the discussion on the origin of numerical abilities.

### (a) Representation

Domain-specific knowledge is pre-specified before learning and must be controlled by the genotype. This view, first endorsed in the domain of language by leading scholars like Chomsky [28] and Pinker [29], has been also applied to the notion of number representation [30,31]. The findings of studies on human newborns and pre-linguistic infants [15,16,32] have often been cited as supporting this type of innatist stance. A related proposal is that there is an innate representation of at least one base quantity, from which the other numbers can be generated through a recursive ‘successor function’ [33]. Together with the extensive literature on numerical abilities in many animal species [34–38], these results converge in promoting an ‘evolutionary perspective’ on the origin of number sense.

### (b) Architecture

The genotype determines architectural constraints (or biases) and the learning algorithms that respond to the environment [27]. Latent structure in the environment—numerosity in the present case—can therefore be acquired by general-purpose learning algorithms. This approach might seem to be at odds with the evidence for numerical competence in early development. However, we show below that this alternative, non-representationalist type of innatism can also adequately address the origin of number sense. It should be noted that the notion of a domain-specific learning mechanism [26] is somewhat intermediate, because it implies the existence of innate learning devices that have specifically evolved for processing numerical information, even if number representations *per se* might not need to be genetically coded [39].

The two approaches markedly differ in how they account for initial numerical competence. Here, we discuss these two hypotheses, building upon computer simulations that investigate sensitivity to numerosity in neural processing systems that are shaped by either evolutionary or architectural constraints. We then investigate the role of sensory experience for refining the ANS, which, regardless of its origin, remains a key issue for understanding developmental changes of number acuity in early childhood.

## 2. Number sense emerges from evolutionary pressure

As noted above, the finding that human infants and many other animal species are sensitive to numerical quantity supports the hypothesis that at least some aspects of number sense might be genetically determined. Though learning and experience clearly play a role in the development and refinement of the number sense [1], this evidence has suggested that these abilities are supported by an ‘evolutionary start-up kit’ [30]. This possibility was explored by Hope *et al*. [40] using artificial life simulations, which exploit behaviour-based selection to capture the impact of evolution. The simulation was built on the hypothesis that quantity comparison emerges from selective pressure to forage effectively [9]: evolving quantity-sensitive foragers would imply the ability to judge quantity and therefore a tendency to ‘go for more’ [37].

**Figure 1.**
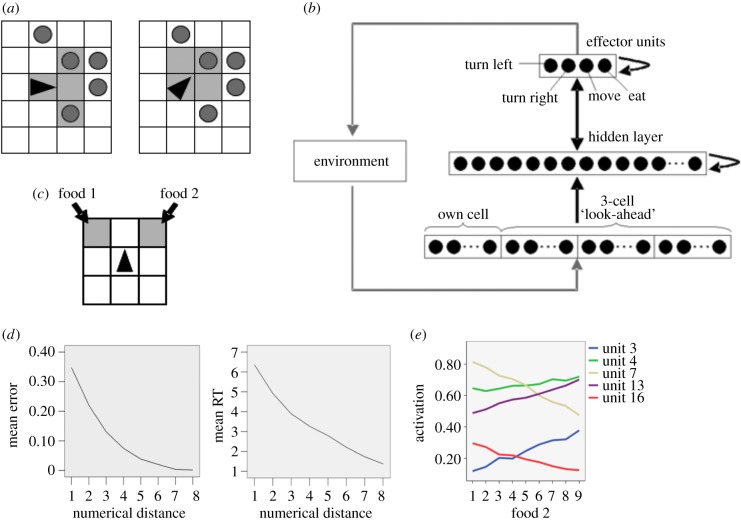
(*a*) Representation of an agent (black triangle) within a section of its artificial ecosystem. In the left panel, the agent is facing right and can sense food (grey circles) in its right and left sensor fields. The right panel shows the same agent after making a single turn to the left: it can now sense only one cell containing food. (*b*) Schematic representation of the recurrent architecture controlling each agent. The sensory layer receives information from the four cells in the agent's field of view, with food quantity in each cell encoded by nine binary neurons. The effector neurons in the motor layer define which action is chosen at each time-step. (*c*) Test trial, where the agent must select between two food sources encoding different numerosities. (*d*) Mean accuracy (left panel) and response time (RT, right panel) as a function of the numerical distance between food quantities. (*e*) Examples of numerosity-sensitive hidden neurons showing monotonically increasing or decreasing response profiles (i.e. summation coding). All panels have been adapted from [40].

The artificial ecosystem used in the simulations is depicted in figure 1*a*. It consisted of a two-dimensional (2D) grid of 100 × 100 cells, each containing a certain amount of ‘food’. Food was randomly distributed and it could take any value between 0 and 9 in each cell. The ecosystem had a fixed population of 200 agents, each controlled by a recurrent, asymmetrically connected neural network (figure 1*b*). The ecosystem evolved by iterative update: each update allowed every agent the opportunity to sense its environment and act. Sensory input to a given agent was defined by its ‘field of view’, which included the current cell and the three cells directly ahead. The information gathered from each visible cell was the food quantity *n*, which consisted in a binary vector with *n* randomly chosen active neurons. A similar coding scheme has been used by Verguts & Fias [41]; however, it is important to emphasize that this coding strategy dispenses with the problem of normalizing object size [24,42]. Finally, each agent possessed a basic repertoire of actions, encoded at the level of the effector neurons: that is, they could turn left or right, move forward or eat.

Importantly, the agents' neural network was shaped by evolution rather than by lifelong learning. The evolutionary process was controlled by a genetic algorithm, which included crossover and mutation operators (for details, see [40]). The agent's genome determined the connection weights and the size of the hidden layer, which were initially set to random values. The goal of the evolutionary process was to promote the emergence of agents that forage in a quantity-sensitive manner, which can be achieved by defining as ‘fitness function’ the rate at which the agent collects food. At each iteration of the ecosystem, two ‘parents’ were randomly selected from the population and the weaker of the two (in terms of fitness value) was replaced by their ‘child’, which was defined by mixing the parents' genomes and applying a small random mutation. As typical in evolutionary simulations, the average efficiency of food collection increased over time and it became high after several million iterations. At that point, the agents' behaviour was tested systematically in terms of their quantity comparison performance. Each agent was removed from the ecosystem and placed into a 3 × 3 test environment (figure 1*c*), where the top left and top right cells contained food of varying quantity. Each trial started with the agent in the centre position, where it could ‘sense’ both quantities, and it was allowed to move until it selected one of the two food cells. This method mimicked that used by Uller *et al*. [37] to capture quantity comparison performance in salamanders. Accordingly, a correct choice was defined as the selection of the larger of the two food values. Each agent in the final population was tested with every combination of food quantities for 50 repetitions, for a total of 3600 trials per agent.

Though many agents performed above the chance level, few agents performed at chance (approx. 20%). The persistence of non-discriminating agents in the population is interesting because it shows that relatively high rates of food collection can be achieved without quantity discrimination, for example, by trading off decision quality in favour of decision speed. Nevertheless, the population included agents that were highly accurate in quantity discrimination.

Notably, accuracy decreased as the two numbers became larger (i.e. size effect) and it was strongly modulated by numerical distance as typically observed in behavioural studies on animals and humans [43]. The distance effect on both accuracy and response time (number of time-steps until selection of the food cell) is shown in figure 1*d*. Moreover, analyses of the internal dynamics of the neural network revealed that the emergent internal representation of quantity had the form of ‘summation coding’ [42,44], whereby neuronal activity increases or decreases monotonically as a function of numerical magnitude (see examples in figure 1*e*). Numerosity-sensitive neurons with this type of tuning property have been found in the lateral intraparietal area of the monkey brain [45]. This format is broadly consistent with the accumulator model of Meck & Church [46], as well as with the ‘summation clusters’ in the neural network model of Dehaene & Changeux [42]. As we will discuss below, the same type of coding also emerges from unsupervised deep learning on images of object sets.

In summary, the simulations reviewed above show that representational innatism is viable in the context of evolutionary pressure. However, these results have two important limitations. First, as noted above, the simulations dispense with the non-trivial problem of extracting numerosity information in a way that is invariant from covarying continuous visual properties (cumulative area, object size, density, etc.). Whether evolutionary simulations embedding a more realistic sensory input would still show the emergence of numerosity representations remains an issue that requires further investigation. Second, these simulations do not account for the ontogeny of numerical abilities. Indeed, the improvement of number acuity observed during early development in both humans [7] and animal species (e.g. in fish, see [47]) suggests that other mechanisms are also at play. The simulations reported in the following section investigate the role of experience-dependent learning in determining the development of number acuity.

## 3. Number sense emerges from architectural and learning constraints

The nature of the mechanisms underlying numerosity perception has been debated for decades [5,6,42,48,49]. However, the advent of a new generation of artificial neural networks, known as deep learning models [50], has provided modellers the unique opportunity to investigate the emergence of high-level visual skills using realistic sensory input [51]. This framework has been exploited by Stoianov & Zorzi [24] to investigate the emergence of a visual number sense. Their simulations, as well as novel simulations described below, are characterized by two key ingredients: generative learning and hierarchical processing. Intuitively, generative learning corresponds to learning by observation; unlike discriminative learning, there is no supervision or reward because the objective of learning is simply to build an internal model by discovering features or latent causes of the sensory information [52]. In other words, there is no task and the neural network does not receive any information about what is in the input (i.e. there is no feedback/supervision signal: all training data are unlabelled). Generative learning becomes particularly powerful when embedded into a hierarchical architecture, where many layers of neurons form a deep neural network [51,53], also known as ‘deep belief network’ [52].

Unsupervised learning in deep neural networks has provided a state-of-the-art and neurobiologically plausible account of how visual numerosity is extracted from real images of object sets [24]. Numerosity emerged as a high-order statistical property of images in a deep network, which learned a hierarchical generative model of the sensory input. The key idea was that numerosity is a statistical invariant of highly variable visual input, and for this reason, it might be encoded as a high-order visual feature (summary statistics) in a deep neural network that simply ‘observes’ images of object sets with variable numerosity. The hypothesis was therefore that numerosity is a latent factor that explains variability in the images of sets of objects. As a result of this unsupervised learning, numerosity-sensitive neurons emerged in the deepest layer of the network, with tuning functions resembling summation coding as observed in the lateral intraparietal area of the monkey brain [45]. The population code provided by number-sensitive neurons in the model was found to be largely invariant to continuous visual properties, and it supported numerosity estimation with the same behavioural signature (i.e. Weber's Law for numbers) and accuracy level (i.e. number acuity) of human adults. Analyses of the emergent computations in the model showed that numerosity was abstracted from lower-level visual primitives through a simple two-level hierarchical process, which exploited cumulative surface area as a normalization signal (also see [54,55]).

In the simulations reported below, we addressed more directly the question of how much sensory experience is necessary for observing number-sensitive behaviour in a deep network. In particular, we pursued the hypothesis that sensitivity to numerosity might emerge in a hierarchical architecture before any sensory experience of object sets, provided that it is endowed with basic visuospatial processing mechanisms. We then investigated how subsequent experience-dependent learning would further shape numerosity representations, thereby leading to the progressive improvement in numerosity discrimination performance.

### (a) Simulating the origin and development of number sense

**Figure 2.**
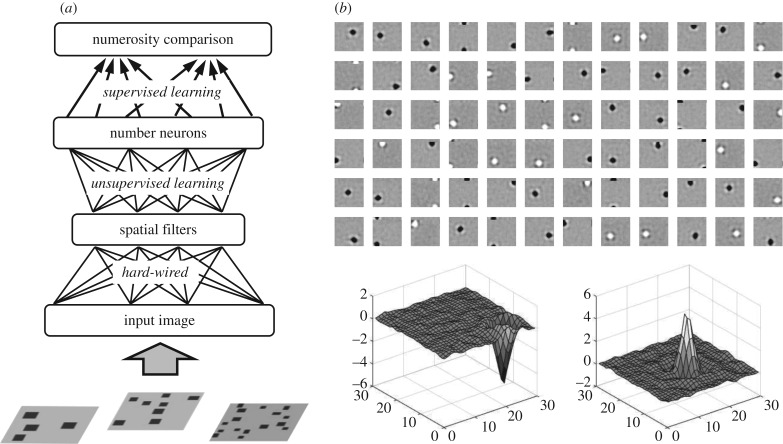
(*a*) Schematic representation of the deep learning model. Stimuli are provided through an input layer, and activation is then propagated through a first hidden layer encoding a set of simple spatial filters (hard-wired connections) and a second hidden layer encoding numerosity information (connections adjusted through unsupervised learning). These internal representations are finally read-out by a response layer to simulate the numerosity comparison task. (*b*) Receptive fields of the spatial filters (on- and off-centre detectors) used in the first hidden layer. Strong, negative connections are represented in black, while strong, excitatory connections are represented in white. Grey colour indicates that connection weight is around zero. A 3D representation of two prototypical off- and on-centre detectors is reported at the bottom.

In our simulations, we used a processing architecture similar to that used by Stoianov & Zorzi [24]. Visual stimuli (see appendix A for details) were provided to the network through an input layer, and activation was then propagated over three additional layers (figure 2*a*). The connection weights of the first hidden layer were fixed in order to encode a set of simple on-centre and off-centre detectors (figure 2*b*). This way learning in the deep network did not start from a completely random configuration, but rather incorporated generic visuospatial processing mechanisms that are likely to be already present at birth [56,57]. The receptive fields of these neurons closely resemble those recorded at early visual processing stages, such as in the retinal ganglion cells and lateral geniculate nucleus of the thalamus [58–60], whose structural and functional development seems to happen independently of sensory experience [61] (also see [62] for a neural network simulation). Note that neurons in the first hidden layer belong to a general visuospatial processing system and cannot explicitly encode numerosity. Location-specific filters were created by mimicking those encoded at the first hidden layer of the original model. These high-frequency spatial filters discretize the visual input and provide a crucial signal to upstream neurons in the processing hierarchy; the latter can compute numerosity by summing the activity of many spatial filters and normalizing it using a signal that encodes cumulative area [24,55]. Note that the key role of high-frequency spatial filtering has also been independently highlighted in a psychophysical model [63].

The connection weights of the second hidden layer were instead randomly initialized, and then gradually adjusted through unsupervised generative learning using the same procedure and training dataset of the original model (see appendix A). Finally, the top layer in figure 2*a* ‘reads out’ the internal representation at the second hidden layer and is trained to map it onto an overt response to carry out the numerosity comparison task. As in the original model, task learning is supervised (see appendix A) and it only requires a simple form of associative learning (such as the delta rule, which is formally equivalent to the Rescorla–Wagner rule in classical conditioning [64]). Read-out accuracy measured at different numerical ratios was used to estimate the model's Weber fraction. Numerosity comparison was assessed before any experience-dependent learning to investigate the initial competence of the model, and then at several time points during unsupervised learning (every 30 epochs) to track the progressive refinement of number acuity.

**Figure 3.**
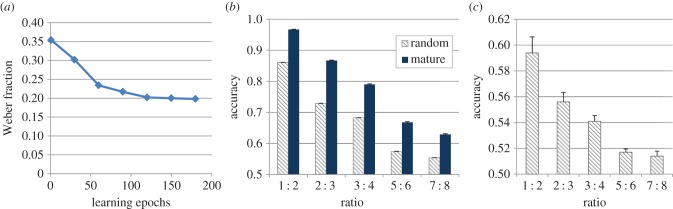
(*a*) Learning trajectory of the model, corresponding to the estimated Weber fraction after every 30 learning epochs. (*b*) Accuracy of the model in the number comparison task as a function of numerical ratio (chance level is at 0.5) when only 25% of the stimuli were used for task learning (supervised training). Performance of the initial network (random) is compared with that of the network following unsupervised learning (mature). (*c*) Performance of the initial network when only 1% of the stimuli were used for task learning. (Online version in colour.)

As shown in figure 3*a*, the initial Weber fraction of the model was approximately 0.35, and then it gradually improved until it converged to 0.20 in the final learning stages. This suggests that, as observed in children [13,14,65], also in the model number acuity undergoes a progressive refinement, reaching a final value that is comparable to that of illiterate humans [66]. The remarkable initial performance achieved by the model is surprising, because the connections of the second hidden layer were random, that is, they were not yet tuned by the sensory experience. This finding therefore suggests that a hierarchical architecture endowed only with basic visuospatial processing at the lowest layer can support a non-trivial level of numerical competence.

However, it should be stressed that, even if the connection weights of the second hidden layer were random at this initial stage, learning the comparison task at the read-out layer involved a consistent amount of experience as well as explicit feedback on the response. In order to assess whether read-out would be possible even when supervised training was significantly reduced, we randomly selected only 25% of the patterns (4600 images out of the 51 200 used in the original model) for learning the comparison task. We note that this reduced amount of feedback is in line with that used in training studies with humans [67,68] and other mammals, such as macaques [69] and dolphins [70]. As shown in figure 3*b*, discrimination accuracy was still remarkably high, especially for large numerical ratios. Crucially, this held also for the network with random connection weights in the second hidden layer. Read-out performance was higher—and approached human performance [67]—after unsupervised learning.

In another set of simulations, we pushed this method to the limit and only selected 1% of the patterns to train the read-out layer, for a total of 184 image pairs. This massively reduced amount of feedback is compatible with that provided in studies involving lower vertebrates (e.g. [71]), which for practical reasons are usually trained using a limited number of trials [72]. As shown in figure 3*c*, read-out from the initial network under this extremely limited feedback regimen still succeeded, especially for the easier ratios. This finding corroborates the hypothesis that even randomly connected deep networks endowed with simple visuospatial processing mechanisms can support numerosity comparison.

### (b) Influence of continuous visual cues on numerosity perception

The remarkable performance of the model, especially before visual experience, raises the recurring question of whether numerosity comparison might in fact be carried out using low-level continuous visual properties as a proxy for discrete numerosity [73–77]. Indeed, even if test stimuli are carefully designed to control for continuous variables, simultaneously controlling all of them is not possible [78].

**Figure 4.**
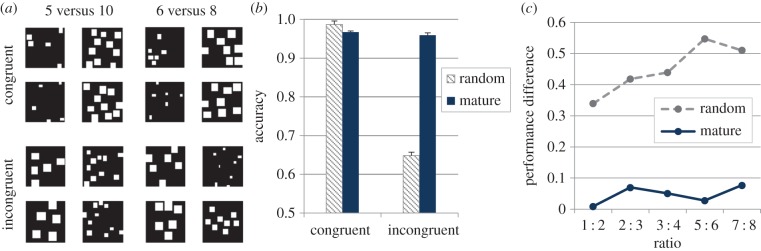
(*a*) Examples of congruent and incongruent stimulus pairs correctly classified by the read-out layer of the initial (random) network. (*b*) Accuracy of initial and mature networks on congruent and incongruent trials (numerical ratio is 1 : 2). (*c*) Cost of incongruency (performance difference between incongruent and congruent trials) for the initial and mature networks as a function of numerical ratio.

To assess the influence of continuous visual cues in the model, we created a set of image pairs in which cumulative area, contour length and individual item size were congruent with numerosity, whereas another set contained image pairs where all these properties were incongruent with numerosity. The model was then tested on these sets, at both the initial and mature stages. Notably, the initial (random) network was often successful even on incongruent trials (examples of correctly classified incongruent trials are shown in figure 4*a*). Nevertheless, congruency had a much stronger impact on the initial network. In particular, as shown in figure 4*b* for the specific ratio of 1 : 2, the mean accuracy of the initial network on congruent trials was much higher (99%) compared with that measured on incongruent trials (65%), whereas accuracy of the mature network was close to ceiling in both cases (97 and 96%, respectively). Moreover, for the initial network, the cost of incongruency on performance increased as a function of numerical ratio (figure 4*c*).

The developmental trajectory of the sensitivity to incongruent visual cues is an important issue for future work, both empirical and computational. However, the high cost of incongruency observed in the initial network is aligned with the finding that both typically developing children [79] and developmental dyscalculics [80] are less accurate on incongruent trials in numerosity comparison. Note that the greater resilience of the mature network to this type of manipulation is not related to the comparison task *per se* (i.e. training of the read-out layer was identical), but it stems from the emergence of more robust representations of numerical information (i.e. invariant to physical appearance) following unsupervised learning on visual sets.

### (c) Emergence of number coding

**Figure 5.**
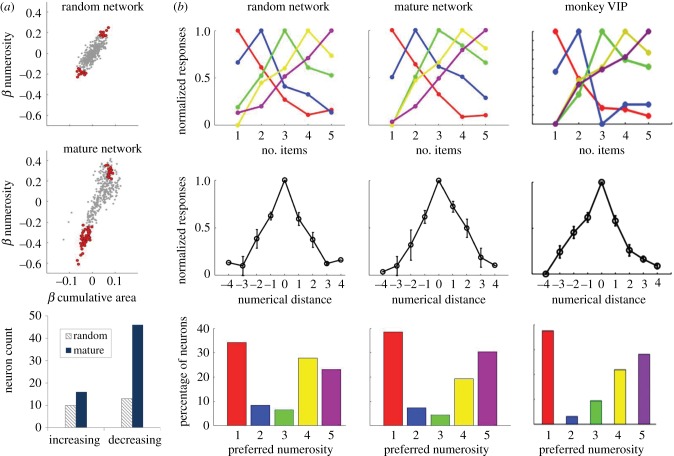
(*a*) Summation coding in the model. The first two scatter plots represent the distribution of numerosity and cumulative area regression coefficients (B) for the initial (random) network and for the trained (mature) network, respectively. The bottom panel shows the count of increasing (positive slope) and decreasing (negative slope) numerosity detectors. (*b*) Numerosity-selective coding in the model compared with neurons in monkey ventral intraparietal area (VIP; neurophysiological data from [81], reproduced with permission). First row: normalized responses averaged for neurons preferring the same numerosity. Second row: response profiles plotted against the numerical distance from the preferred numerosity. Third row: frequency distributions of preferred numerosity in the population of numerosity-selective neurons.

We replicated the regression analysis performed by Stoianov & Zorzi [24] to investigate whether there were neurons at the second hidden layer of the network (see figure 2*a*) specifically tuned to numerosity information, rather than to cumulative area (see appendix A). In particular, the response profile of *numerosity-sensitive* neurons (‘numerosity detectors' in [24]) should be invariant to cumulative surface area: this is indexed by a large absolute value for the numerosity regression coefficient and a small value for the cumulative area coefficient. As shown in figure 5*a*, numerosity-sensitive neurons were found even in the initial network (*n* = 23), although their number significantly increased following learning (*n* = 62). These response profiles can be considered as a form of summation coding [42,44]: a positive value of the coefficient indicates that activation increases monotonically as a function of numerosity, while a negative slope indicates that it monotonically decreases. As can be noted in figure 5*a*, the response strength of numerosity-sensitive neurons (indexed by the regression coefficient) increased as a result of unsupervised learning. Moreover, the bottom panel of figure 5*a* shows that the impact of learning was mostly related to the percentage of neurons negatively tuned to number (almost a threefold increase).

One limitation of the regression analysis, however, is that it can only detect monotonic response profiles. Many empirical studies have shown that the neuronal code for number in the primate cortex also relies on neurons selectively tuned to specific numerosities (for review, see [12]). *Numerosity-selective* neurons respond most strongly to one preferred number, but they also respond to a lesser extent to adjacent numbers, thus exhibiting a bell-shaped, nonlinear response function. These neurons have been observed in numerically naive monkeys [81], suggesting that this more sophisticated form of encoding might spontaneously emerge early during development. This way of coding numerical information also seems to have evolved independently in vertebrate brains with very different anatomies [82], thereby supporting the hypothesis of convergent evolution. We adopted the procedure used by Viswanathan & Nieder [81] to assess whether numerosity-selective neurons can also be found in our model. A two-factor analysis of variance (ANOVA) was used to select neurons whose activation was modulated by numerosity information but not by cumulative area (see appendix A). Individual tuning curves of the neurons with the same preferred numerosity were then pooled to compute average response profiles. Interestingly, numerosity-selective neurons were found both in the initial (*n* = 108) and in the trained (*n* = 135) networks, with average response profiles (first two columns in figure 5*b*) strikingly similar to those observed in the ventral intraparietal (VIP) area of the monkey brain (last column in figure 5*b*). The distribution of neurons across the range of numerosities closely mirrored the empirical data [81], especially for the mature network.

Though summation coding and numerosity-selective coding might characterize distinct neuronal populations, as assumed in popular computational models (e.g. [41,42]), one potential caveat is that the response profiles of numerosity-selective neurons at both extremes of the tested range are in fact monotonic, and might thus be considered as a form of summation coding. Moreover, computer simulations have shown that a pool of summation coding neurons can exhibit numerosity-selectivity at the population level [83]; a more precise characterization of individual tuning functions is the focus of ongoing research [84]. The present findings suggest that even basic visuospatial filtering combined with a random projection is sufficient for exhibiting numerosity-selectivity. Nevertheless, number coding in the model was significantly refined by visual experience, as suggested by the increasing response strength of numerosity-sensitive neurons and by the reduced impact of continuous visual cues (as shown in section 3b). Unsupervised learning therefore appears to fine-tune the response profiles both at the single neuron and at the population levels.

## 4. Conclusion and future directions

During the last decades, an impressive amount of empirical research has shown that non-verbal numerical abilities are widespread within the animal kingdom. These findings have been usually interpreted as evidence for evolved, biologically determined numerical capacities across unrelated species, thereby supporting a ‘nativist’ stance on the origin of number sense. In this article, we have framed the problem of the origin of number sense within an ‘emergentist’ perspective [25], whereby the neural processing systems that implement perception and cognition might be shaped by a variety of constraints, which include evolutionary, architectural and learning biases.

We have shown that, although numerical abilities can be supported by domain-specific representations emerging from evolutionary pressure (as in the simulations with the artificial ecosystem), numerical representations need not be genetically pre-determined as such. Indeed, they can also emerge from the interplay between innate constraints (e.g. simple visuospatial processing embedded into a hierarchical architecture) and domain-general learning mechanisms (e.g. unsupervised learning of an internal model of the environment). Our computer simulations demonstrated that multi-layer (deep) neural networks endowed with only basic high-frequency spatial filters exhibit a remarkable performance in numerosity discrimination. Moreover, following exposure to sets of visual objects, the network gradually refined its internal representation of numerosity, thereby improving discrimination performance up to the level of adult human observers. Thus, our simulations are the proof-of-concept that a form of innatism based on architectural and learning biases is a viable approach to understanding the origin and development of number sense across species.

It should be noted that one possible issue with connectionist models is that several modelling choices, such as the format of the input and output representations [85] or the particular choice of learning algorithm [86], may have a crucial impact on the model's behaviour. One strength of the simulations presented in this article is that we made virtually no assumptions about the input/output representation format (e.g. the visual stimuli were real images, encoded at the pixel level) and the learning algorithm (e.g. internal representations in the model emerged from probabilistic, generative learning on the sensory signals, in line with modern theories of cortical learning [87–89]). Notably, a visual number sense emerges even when the deep network is exposed to a more ecological set of images, that is, when the size and the displacement of the items is obtained by segmenting objects in natural scenes (WY Zou, A Testolin, JL McClelland 2017, submitted). However, it is still to be shown whether a further refinement of numerical representations could be boosted by ‘recycling’ [90] generic visual features learned from natural images, as recently shown in the domain of visual letter recognition [91].

Key issues remain to be addressed in future research. For example, it should be stressed that our behavioural task was implemented by training a supervised read-out layer on the internal representations developed by the deep network. Though this is compatible with common experimental procedures, where explicit feedback is provided to the subjects [67–70,72], many studies carried out with newborns and infants are instead based on habituation paradigms [15–17]. There have been concrete proposals about how to simulate habituation tasks using artificial neural networks [92,93], but they have not yet been exploited in the field of numerical cognition.

A promising research direction would also be to more carefully investigate how the basic visuospatial filtering implemented in the first hidden layer of our model relates to spatial acuity in newborns. In our simulations, this early processing stage was fixed for simplicity, but it would be more realistic if spatial acuity also could change during development [57]. Although in principle the refinement of early processing stages should still be supported by unsupervised learning (i.e. it should happen independently from explicit numerical training), simulating the joint development of all hidden layers of a deep neural network is challenging because it requires a progressive learning algorithm (WY Zou, A Testolin, JL McClelland 2017, submitted).

Another interesting question relates to the computational properties of random matrices. How is it possible that random projections, such as those implemented at the deepest layer of our ‘initial’ network, create internal states that can be meaningfully read out by a supervised classifier? Although the advantages of transforming input data using random mappings have been explored in several machine learning algorithms [94,95], a parallel with neurocognitive models has not yet been clearly established. A mathematical motivation for the surprising effectiveness of random projections is based on the Johnson–Lindenstrauss theorem, which suggests that good representations for classification and discrimination of visual objects can indeed be obtained by dot products of the image with random templates, because the latter provide a quasi-isometric embedding of images [96]. However, understanding how this theory extends to the case of visual numerosity, which implies a different type of variability in the sensory input with respect to the case of object recognition, is still an uncharted territory.

Finally, one of the most pressing questions to be addressed in future research is whether the generic processing and learning constraints incorporated in our model would suffice even for developing more sophisticated types of numerical abilities, such as those underlying symbolic quantification and arithmetic, which likely require cultural mediation [97] and whose acquisition profoundly reshapes our brain [98]. Mathematical thinking is a hallmark of human intelligence and one of the most impressive achievements of human cultural evolution, as well as a major target of educational efforts; a deeper understanding of its neurocomputational foundations is therefore the key to the possibility of formally assessing the impact of different learning strategies both for normal children and in remedial treatment of mathematical learning disorders.

## Data accessibility

The data and complete source code of our model is available for download (http://ccnl.psy.unipd.it/research/deeplearning).

## Appendix A

**(a) Visual stimuli**

Images containing a variable number of items were created using the same procedure as described by Stoianov & Zorzi [24]. In particular, a certain number of white rectangles (ranging uniformly between 1 and 32) were randomly placed on a black display of size 30 × 30 pixels. Cumulative area of the items uniformly varied among eight levels (32, 64, 96, 128, 160, 192, 224, 256). Overall, 200 different images were created for each combination of number/cumulative area, for a total of 51 200 patterns.

**(b) Training and testing procedures**

On- and off-centre detectors in the first hidden layer had approximately the same receptive field size (mean diameter 6.3 pixels, standard deviation 0.5). Unsupervised learning in the second hidden layer was implemented by training a restricted Boltzmann machine (RBM) on the hidden unit activations of the first processing layer using the contrastive divergence algorithm [99]. The RBM had 74 visible neurons (corresponding to all on- and off-centre detectors) and 400 hidden neurons. Connection weights were randomly initialized according to a Gaussian distribution with mean 0 and standard deviation of 0.01. Learning was performed on graphic processing units [100] for a total of 180 epochs, using a learning rate of 0.1, a momentum coefficient of 0.9 and a weight decay factor of 0.0004. The supervised read-out layer was trained using an efficient implementation of linear associative learning (‘pseudoinverse method’ [101]). In the simulation of the developmental trajectory of number acuity (figure 3*a*), the read-out layer received the pattern of activation of neurons in the second hidden layer (elicited by a given image) and was trained to assess the numerosity against a reference value of 12. Input numerosities varied between 4, 6, 8, 9, 16, 18, 24 and 32. Comparison accuracy at different numerical ratios was used to estimate the model's Weber fraction (as in [24]). In all subsequent simulations, the read-out layer simultaneously received the pattern of activity elicited by two different input images and was trained to assess which of the two contained the larger numerosity (as in [54]). This constitutes a more realistic approximation of the experimental procedure adopted in empirical studies, where the comparison typically involves two visual sets rather than a fixed internal standard, and it is appropriate for assessing the influence of continuous visual properties (see section 3b). Input numerosities were selected with the aim of creating five different levels of numerical ratio: 1 : 2 (5 versus 10; 6 versus 12; 7 versus 14); 2 : 3 (6 versus 9; 8 versus 12; 10 versus 15); 3 : 4 (6 versus 8; 9 versus 12; 12 versus 16); 5 : 6 (5 versus 6; 10 versus 12) and 7 : 8 (7 versus 8; 14 versus 16). Response variability was obtained by replicating 10 times the supervised training of the read-out layer.

**(c) Number coding**

The regression analysis was carried out using the method described for the original model [24]. A linear regression was performed on the activation profile of each hidden neuron in the second hidden layer, using numerosity and cumulative surface area as predictors. Numerosity varied between 1 and 32, while cumulative area varied across five levels (96, 128, 160, 192, 224 pixels). All variables were normalized between 0 and 1 and both predictors were logarithmically transformed. The criterion for a neuron to be classified as a numerosity detector was that the regression explained at least 10% of the variance and the regression coefficient of the cumulative area had an absolute value smaller than 0.1 (see [24, electronic supplementary material]). Numerosity-selective neurons in the second hidden layer were found using the method described in [81]. A two-way ANOVA with numerosity and cumulative surface area as factors was performed for each neuron to identify those with activity significantly modulated only by numerosity (threshold criterion: *p* < 0.01; other main effects and interactions not significant; see [81]). Numerosity varied between 1 and 5, while cumulative area varied across five levels (96, 128, 160, 192, 224 pixels). All individual response profiles were normalized between 0 and 1. The average response profiles for numerosity-selective neurons were computed by first grouping the neurons according to their preferred numerosity (indexed by the maximum activation value) and then averaging the individual tuning curves.
